# Conditional Cooperativity of Toxin - Antitoxin Regulation Can Mediate Bistability between Growth and Dormancy

**DOI:** 10.1371/journal.pcbi.1003174

**Published:** 2013-08-29

**Authors:** Ilaria Cataudella, Kim Sneppen, Kenn Gerdes, Namiko Mitarai

**Affiliations:** 1 Niels Bohr Institute/CMOL, University of Copenhagen, Copenhagen, Denmark; 2 Centre for Bacterial Cell Biology, Institute for Cell and Molecular Biosciences, Newcastle University, Newcastle, United Kingdom; University of Virginia, United States of America

## Abstract

Many toxin-antitoxin operons are regulated by the toxin/antitoxin ratio by mechanisms collectively coined “conditional cooperativity”. Toxin and antitoxin form heteromers with different stoichiometric ratios, and the complex with the intermediate ratio works best as a transcription repressor. This allows transcription at low toxin level, strong repression at intermediate toxin level, and then again transcription at high toxin level. Such regulation has two interesting features; firstly, it provides a non-monotonous response to the concentration of one of the proteins, and secondly, it opens for ultra-sensitivity mediated by the sequestration of the functioning heteromers. We explore possible functions of conditional regulation in simple feedback motifs, and show that it can provide bistability for a wide range of parameters. We then demonstrate that the conditional cooperativity in toxin-antitoxin systems combined with the growth-inhibition activity of free toxin can mediate bistability between a growing state and a dormant state.

## Author Summary

The effectiveness of antibiotics on many pathogenic bacteria is compromised by multidrug tolerance. This is caused by a small sub-population of bacteria that happen to be in a dormant, non-dividing state when antibiotics are applied and thus are protected from being killed. These bacteria are called persisters. Unraveling the basic mechanism underlying this phenomenon is a necessary first step to overcome persistent and recurring infections. Experiments have shown a connection between persister formation and the battle between a toxin and its antitoxin inside an *E. coli* cell. Toxin inhibits the cell growth but is neutralized by the antitoxin by forming a complex. The proteins also regulate their own production through this complex, thereby forming a feedback system that controls the growth of the bacterium. In this work we provide mathematical modeling of the feedback module and explore its abilities. We find that the auto-regulation with reduced growth associated with free toxins allows the cell to be bistable between two states: an antitoxin-dominated, normal growing one, or a dormant one caused by the activity of the toxin. The latter can be the simplest description of persister state. The toxin-antitoxin system presents a powerful example of mixed feedback design, which can support epigenetics.

## Introduction

Many bacteria and archaea have multiple Toxin-Antitoxin (TA) loci [1], where the toxin normally inhibits cell growth, while the antitoxin neutralizes the activity of the toxin by forming a tight TA complex. One of the known functions of TA loci is to respond to nutritional stress [2], namely, toxins are activated upon nutritional starvation and slow down the rate of translation. When cells are under normal fast growth conditions, on the other hand, the majority of the cells will be in the antitoxin-dominated state, such that toxin activity is fully inhibited.

It has been found that many bacterial TA loci are auto-regulated at the transcriptional level by a mechanism called “Conditional Cooperativity” (CC) [3], where the transcription factor can bind cooperatively to the operator only if the concentrations of two different proteins satisfy a certain stoichiometric ratio. CC was quantitatively studied in one of the *Escherichia coli* TA loci, *relBE* [3]–[6]. Here the two proteins, the toxin (mRNase) RelE and the antitoxin RelB, are encoded by the same operon, which is negatively auto-regulated. The tight dimer 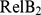 is a weak transcriptional auto-repressor, but this repression is strongly enhanced by the presence of RelE and becomes strongest at 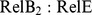 ratio 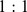. Over-expression of RelE above twice of 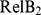, though, will result in an abrupt de-repression of the promoter. This unique behavior is a consequence of formation of alternative hetero-complexes of RelB and RelE; 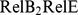 and 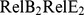. Two 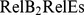 bind to the promoter site cooperatively to repress the promoter strongly, while 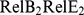 does not bind to the promoter.

Interestingly, all plasmid and chromosome-encoded TA loci investigated are found to be regulated by CC so far, including *relBE* of *E. coli* [3], [4], *vapBC* of *Salmonella enterica* [7], *phd/doc* of plasmid P1 [8], [9] and *ccdA/ccdB* of plasmid F [10]. This suggest that CC is a common feature for TA loci.

In our previous work, we have explored the function of CC in the starvation response of the RelBE system, and showed that CC prevents random toxin activation and promotes fast translational recovery when starvation conditions terminate. However, to reproduce the full dynamics of the starvation response, we took into account details of the RelBE system, which made the model rather specific to it. The primary purpose of this paper is to construct a simple mathematical model that demonstrates the functions of CC in a more general perspective.

TA loci have been suggested to be involved in persister formation [11]–[16]. When an antibiotic is applied to a growing bacterial population, the majority of the bacteria are killed. However, a very small fraction of them survives and re-grows after the antibiotic is removed. If the progeny of the bacteria is again sensitive to the same antibiotic, they are called persisters, in contrast to the resistant bacteria that have acquired resistance to antibiotic by mutation. Persisters are genetically identical to the sensitive cells, but believed to be in a non- or slow-growing, dormant state. Since the majority of antibiotics interferes with the cell growth and division process, cells can survive if they grow slowly or not at all.

The exact molecular mechanism underlying persistence is not fully understood. However, it has been found that mutations in *hipAB* genes severely increase the level of of persisters formation. Interestingly *hipAB* is one of the TA loci in *E. coli* [11], [13], [14]. In addition, recent experiments [15] showed that removal of 10 mRNase-encoding TA loci reduced the persister fraction significantly. These observations strongly suggest that TA loci are important factors for persister formation.

One of the possible explanations is that stochastic activation of the toxin will slow down cell growth, resulting in a dormant state. This will be possible if the TA locus dynamics exhibits bistability, where a cell can be either in the antitoxin-dominated state that ensures the growth or in the toxin-dominated state that inhibits the growth. This viewpoint is also consistent with the observation that the persister state can be described as a metastable state with a constant stochastic switching rate to and from normal growing state [12].

This idea was theoretically pursued by Lou et al. [17] with a simple mathematical model that did not take CC into account. They concluded that, for bistability to be achieved, high cooperativity (Hill-coefficients 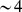) is necessary, both in transcriptional auto-regulation of the TA operon and in the free toxin activity.

In this paper, we explore the basic features of CC as a regulation mechanism mediated by heteromer formation. We demonstrate that CC provides bistability in a simple feedback motif in a wide range of the parameters. We then construct a simplified model of TA system regulation and demonstrate that CC with growth rate-mediated feedback via toxin activity can provide the bistable alternatives between the antitoxin-dominated and the toxin-dominated states.

## Results

### Conditional regulation

#### Complex formation

**Figure 1:**
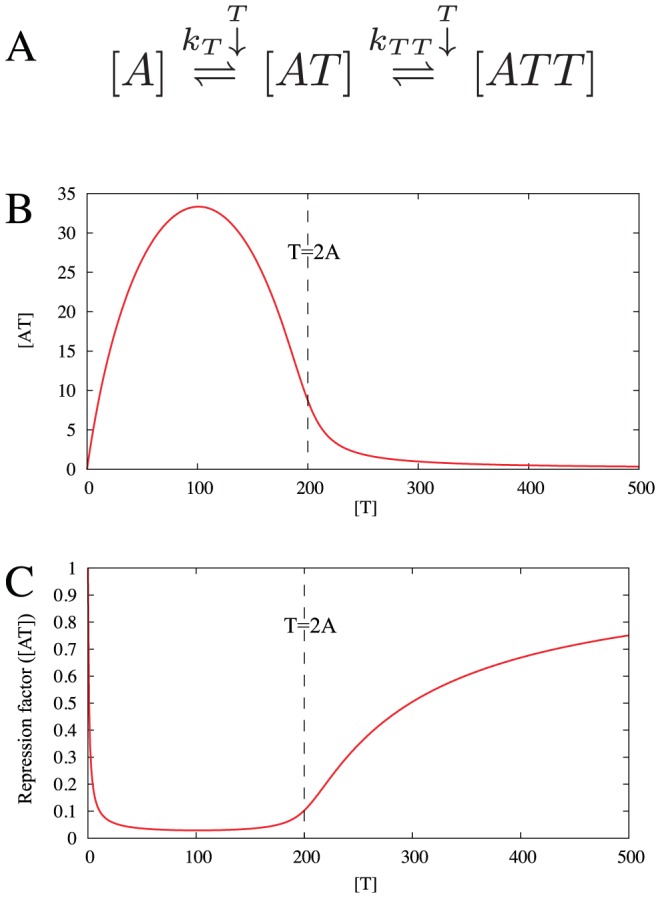
Heterocomplex formation in a TA system. (A) Reaction scheme of the heterocomplex formations, implying that the active complex [AT] is constrained by through 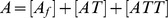 and 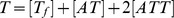 with complex concentrations expressed by eq. (2). (B) Concentration of AT heteromers for a fixed value of 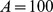 as a function of 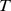 with 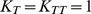. Note that it has a peak at 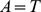. In the strong binding limit of 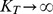 with 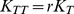 (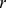 kept constant), 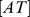 for 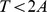 is given by 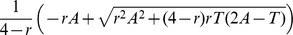 for 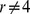 and 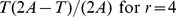, where 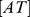 always has a peak at 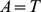. In this limit, 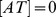 for 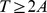. (C) The behavior of 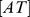 shown in (B) is reflected in the behavior of the repression factor 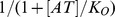 as a function of 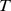, calculated for fixed 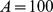, and dissociation constant for AT-DNA binding 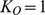.

We examine a simplified system, where protein A and T can form two kinds of heteromers, AT and ATT (Fig.1A):
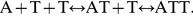
(1)Here, we assume that AT is the active molecule that act as a transcriptional repressor, whereas free A, free T, and ATT are not active in transcriptional control. This is a simplification of the transcriptional regulation by RelBE, where RelB_2_ corresponds to one A, while RelE corresponds to one T.

The amount of active molecule 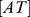 shown in Fig. 1 is determined from total 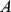 and 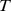 distributed among complexes 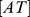 and 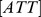 according to
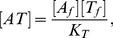
(2)

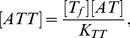
(3)Here 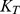 and 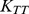 are the dissociation constants for AT and ATT, respectively, whereas the concentration of free A (T) is denoted 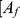] (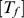).

Fig. 1B shows 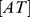 as a function of 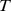 for fixed 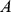, pinpointing that when 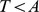, 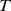 is limiting the amount of AT, while 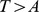 implies that a substantial fraction of 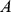 is sequestered in the ATT complex. For 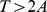, ATT formation sequesters nearly all AT and 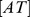 drops sharply to a value close to zero. This last transition can be ultrasensitive, provided that the binding between AT and ATT is strong, 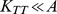. For RelB-E system the binding is indeed very strong, with a measured 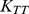 in the nanomolar regime [6]. A sequestration-mediated ultra-sensitivity is also known in small RNA regulation [18]–[21] as well as in transcription factors [22]–[25]. In the present case, just a factor two difference in 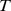 around 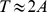 can change 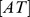 dramatically.

This ultra-sensitivity is reflected in the promoter activity behavior, that shows a sharp de-repression occurring at 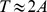 (Fig. 1C), where 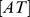 drops. Another unique feature of CC is its non-monotonicity, and an associated derepression for small 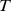 because 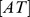 is small, see Fig. 1B,C.

Note that Fig. 1C does not include possible cooperativity in AT-DNA binding. The unique characteristics of CC, ultra-sensitivity by sequestration and non-monotonicity, do not require this cooperativity. For simplicity, therefore, we focus on regulation by AT without cooperativity, and we call it “conditional regulation” (CR), rather than CC. Of course, adding cooperativity will make the response even sharper, and the following results hold for the cooperative case, too.

#### Bistability in a simple feedback motif

We now study production of T repressed by AT, while 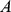 is fixed. The regulatory circuit is described by
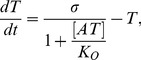
(4)where 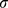 is the maximum production rate of T, and 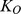 is the dissociation constant of AT molecule to DNA. We assume that total A can be controlled and maintained at a steady state by a AT independent promoter. In this subsection, we take the lifetime of T to be the time unit and set 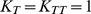 for the dissociation constants, thus measuring concentrations of AT and ATT in units of their mutual binding strength. Further, focusing on CR, we assume that there is no cooperativity in binding of AT to promoter.

**Figure 2:**
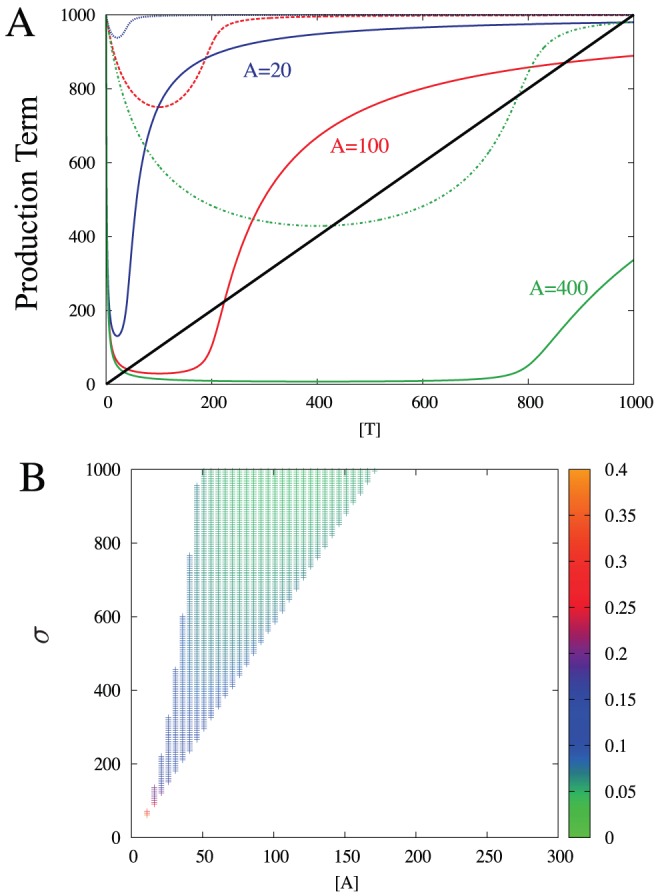
Conditional regulation of T with fixed A concentration. (A) Production term of eq. (4) as a function of 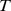 for 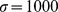, for 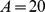 (blue line), 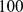 (red line), and 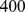 (green line). The solid lines represent 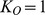 case, and the dashed lines represent 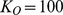 case, where 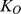 is the dissociation constant for the binding of AT-DNA. (B) Region in the parameter space (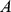, 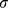) that shows bistability for 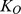 = 1. The color of each bistable point represents the ratio between the low-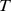 fixed point and the high-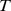 fixed point.

Fig. 2(A) shows the production term of eq.(4) as a function of 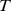, for three different values of 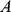 with each of them two different values of 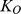. The repression is always strongest at 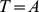, and sharp de-repression happens at 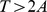 for all the cases. The higher 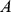, the more 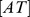 will present when 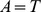, resulting in stronger repression at 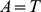 for larger 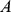. The AT-DNA dissociation constant 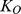 also contribute to the repression strength.

The thick black line represents the degradation term in eq. (4), and the intersection between this and the production gives the steady state values of 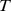. For small 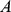 ( = 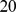) with 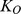 = 1, there is only one crossing, happening at a relatively high value of 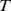 (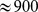.). At intermediate 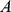 (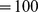), there are two stable fixed points and one unstable fixed point in between (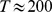), reflecting a bistable system. At high 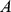 (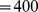), the high 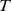 fixed point vanishes and the system settles at a monostable state with low 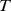. We have also analyzed the systems systematically for weaker repression, i.e. higher values of 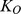, and again found bistability provided that 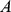 (and thus 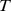) is increased accordingly.

In addition, the non-monotonicity of the CR has a striking implication in regulation at low T values: It guarantees that the low (uninduced) 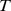 steady state value has finite amount of 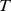 that is maintained at a level nearly independent of 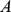 (Fig. 2A, compare 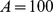 and 400 with 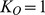.). This is an important feature for TA system in terms of the starvation response, as discussed later.

Remarkably, the system exhibits bistability without cooperative binding to DNA. In the TA system the cooperativity is instead provided by the ultrasensitive de-repression at T = 2A that is facilitated by a very strong protein-protein binding [22]–[25]. This bistability is seen in a wide range of 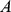 and 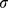 values as shown in Fig. 2(B). The larger 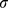 and 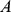, the high-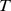 steady state value increase proportionally, while the low-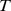 steady state value remains practically unchanged. Thus, as externally imposed 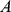 is increased, the model predict a larger contrast between the two steady states. If the binding to DNA is cooperative, the de-repression at ATT formation becomes even sharper, thereby favouring bistability.

We have also studied other possible motifs, where either T or A is repressed or activated by AT complex (data not shown). For example we found that if AT activate A while 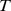 is kept constant, one can obtain bistability between a high 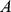 state and a low 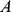 state in a wide range of parameters. This bistability is again supported by the ultrasenstivity of AT sequestration, as 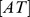 increase sharply with increasing 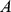 around 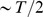.

### Simple model of persister formation

In this section, we construct a simple model of TA activity control with CR, a model aimed at capturing the central features of persister formation. We use the RelBE system as a reference because the molecular interactions and parameters are best known here. The reference parameters are listed in Materials and Methods.

In RelBE [6], the antitoxin RelB and the toxin RelE are encoded by the same operon, and transcriptionally auto-regulated by CC. RelE is metabolically stable, and its concentration decreases only by dilution due to cell division (generation time ∼30 min in log phase growth in rich medium). On the other hand, RelB is actively degraded by protease Lon, resulting in its very short half-life of 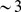 min. In spite of this, the RelB concentration in a normally growing cell is about 10 times of that of RelE [4], suggesting that the RelB mRNA is translated about 100 times more often than RelE mRNA [6].

**Figure 3:**
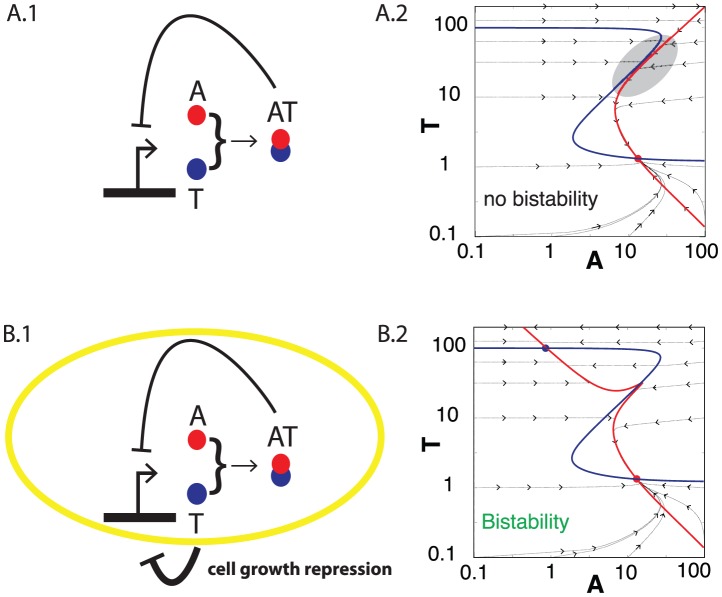
TA system with CR without and with feedback through free toxin activity. (A.1) Schematic representation of the genetic circuit described by eq. (5) for TA system with CR, without considering toxic activity of free T. (A.2) Null-clines for eq. (5). Blue line represents 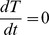, and red line represents 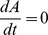. For comparable values of A and T the two null clines become parallel and does not cross, as shown in the area highlighted in grey, i.e. the system does not show bistability. The parameters used are listed in Table 1 in Materials and Methods. Dashed lines with arrows show the flow to the fixed point. (B.1) Schematic representation of the genetic circuit described by the model (6) and (7). (B.2) Null-clines for the system of eqs. (6) and (7) with 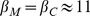. Blue line 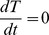, Red line 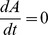. Dashed lines with arrows show the flow to the stable fixed points.

This situation is depicted in Fig. 3A1. Since both toxin T and antitoxin A are regulated by the same promoter, the corresponding equations apply:
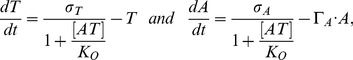
(5)where 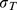 and 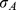 are the maximal production rate for 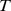 and for 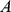, respectively. The dilution rate of 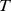 is given by cell division, and is taken as a unit rate, while 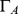 is the active degradation rate of 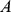.

This motif, however, cannot exhibit bistability. Fig. 3A2 shows example null-clines, which have only one stable fixed point at the antitoxin dominated state. We performed parameter scan spanning from 1/8 to 8 fold relative to the values used for Fig. 3A2, but did not find any combination of parameters that gives bistability, even if we allow cooperative binding of AT to DNA with Hill coefficient 2 (data not shown). This absence of bistability is due to A being regulated identically to T. Accordingly, the de-repression of the promoter around 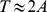 increases not only the toxin production but also the antitoxin production, and the latter is so large that the system remains in the antitoxin-dominated state.

When we include the activity of free toxin on cell growth, however, the model system can show bistability. This is because the toxin-induced arrest of cell growth prolong lifetime of T, while leaving A being degraded by Lon at a high rate. The mathematical formulation of this extended model is
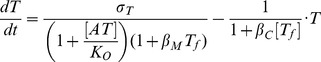
(6)

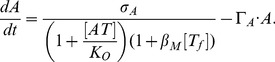
(7)expressing that 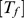 reduces all protein production, and accordingly also decreases the dilution by cell growth. 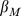 represents the reduction of protein expression per free toxin (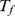) molecule, and 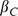 represents the growth inhibition per free toxin molecule. Notice that 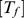 does not influence degradation of A, because it is anyway so unstable that cell division hardly affects its concentration.

These terms correspond to the growth-rate dependent feedback [17], [26], [27]. The reduction of the protein production (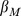 term) can account for both direct activity of free toxin to TA locus and the global slowdown of the transcription rate due to change of physiological conditions [26]. Comparison of the present model with the steady state growth data in Ref.[26] is given in Text S1. We expect 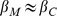 because the slowing down of the growth rate is due to the global slowing down of the protein production. At the same time, there can be some quantitative difference because 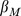 may include the effect specific to the TA locus.

The growth-rate reduction mediated by T constitutes a positive feedback [17], [26], [27] on T accumulation, which is essential for bistability and persister formation. The term with 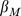 reduces the production of both antitoxin and toxin, and thus overall weaken the ability to maintain the bistability. Note that 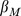 primarily influences the transition state from A to T dominated state, because the reduction of production targets the short lived A protein first.

Fig. 3B1 examines eqs. (6)–(7) with parameters extracted from the RelBE system [6] (see the figure caption of Fig. 3). The null-clines in Fig. 3B2 are from the 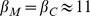 case, exhibiting two stable fixed point, one at the antitoxin-dominated state (the low-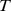 state, 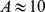, 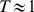) and another at the toxin dominated state (the high-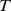 state, 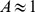, 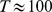). Note that the antitoxin dominated state has almost the same concentrations as the stable fixed point in Fig. 3A2 with 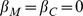. The antitoxin dominated state scarcely depends on 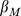 and 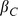, since there is almost no free toxin (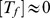) in the antitoxin dominated state.

**Figure 4:**
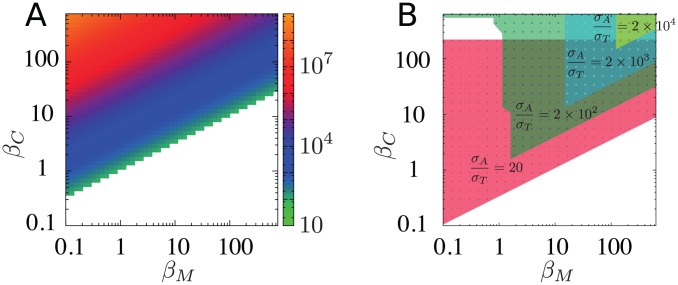
The state diagram of the bistability. Colored region represents the combinations of (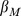, 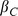) that makes the system bistable. (A) Reference parameters in table 1 are used except for 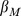 and 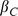. The color code represents ratio between 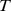 dilution rate calculated upon the low-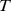 steady state and the high-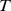 steady state, 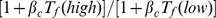. (B) Bistable region for various values of 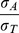, with 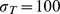. The remaining 6 parameters are fixed to the reference values. The shaded regions represent the areas in the 2D parameters space 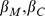 that show bistable behavior.

Figure 4A shows the ratio between the 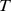 dilution rates at the low and high 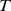 steady state, 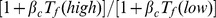. The figure illustrates that our model predicts bistability for a wide range of parameters, and further that this bistability is indeed governed by the increase in cell generation parameterized by the 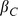 term. For too large 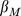 the bistability is counteracted because the toxin production is reduced too much by free toxin to accumulate enough for the stable high toxin state. Remarkably, for proportional reduction of protein production and increased cell generation, 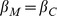, the model predicts bistability for all 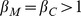.

We also studied the robustness of the bistability against parameter change. One of the most crucial parameters for the bistability is the ratio 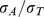, because this determines the difference of the concentration of 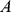 and 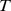. We therefore varied 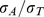 with keeping 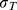 constant, and searched for the bistable regime in 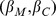 space. The rest of the parameters are kept same as those used in Fig. 4A. Only 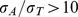 is considered, because lower ratios prevent antitoxin domination due to its 10 times higher degradation rate. For rather small 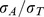 (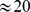), too large 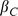 makes the anti-toxin dominated state unstable, because very small amount of free toxin is enough to activate the positive feedback to toxin via the growth rate. With even larger 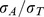, stronger feedback is needed to stabilize toxin-dominated state, reflected in larger values of 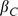 and 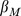.

We further performed scanning of other parameters. We fixed one parameter at a time and sampled the rest of the parameters randomly to test 1000 samples in logarithmic scale within the range between 1/8 to 8 fold of the reference values. We then systematically changed the fixed parameters between 1/8 to 8 fold and repeated the procedure, to see the effect of the parameter. We found that 20% to 80% of the samples showed bistability. The detailed results are given in Text S2. We also explored the effect of the dissociation constant 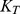 and 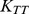 more intensively, by changing 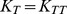 from the reference value to 64 fold, since they describe the sharpness of the CR and this is expected to influence the bistability. We find that the number of bistability parameter sets decreases gradually with the fold change of 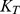 and 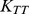. Details are given in Fig. S4.

## Discussion

Using known parameters for the RelBE system in *E. coli*, we constructed a minimal model for TA activity, combining conditional regulation with a feedback from free toxin to the cell growth. It was demonstrated that this model shows bistability for a wide range of parameters, with a stable state corresponding to the antitoxin-dominated, normal growing state, and another metastable state corresponding the toxin dominated state, potentially corresponding to the persister state.

Noticeably, the model eqs. (6)–(7) did not rely on details of the molecular mechanisms of how the toxin works, and therefore the model is not limited to the RelBE system. The important assumptions are: (i) The TA system is conditionally regulated, (ii) toxins are stable and diluted mainly by cell division, while antitoxins are metabolically unstable, and (iii) free toxins reduce the productions of proteins and hence cell growth. All the conditions are satisfied in the TA loci that are confirmed to be regulated by conditional cooperativity [3], .

Our simple model pinpoints minimal ingredients for obtaining a persister state, but did not include stochastic production and/or degradation, and therefore cannot address the switching rates. In order to understand stochastic persister formation in *E. coli*, just performing stochastic simulation of the present motif is not enough, because the frequency of persisters depends on multiple parallel TA systems. In *E. coli*, 11 simultaneously interfering TA systems maintain a probability of persisters to be about 0.01%, while this probability is changed substantially first when about 50% of the TA systems is removed [15]. This clearly suggests that the interference of parallel systems has a strong influence to the switching behavior. Furthermore, comparing the stochastic simulations with the experimentally observed frequency of persisters requires a knowledge of the underlying distribution of the 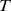 expression levels and corresponding growth rates in the cell population. It is not a simple task when the single cell growth rate depends on 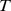 expression levels, because it feedbacks to the frequency of the cells as pointed out by Nevizhay et al. in [28]. In addition, it has been suggested that there is a strong link between the activation of the protease Lon and the TA-mediated persister formation, through the increase of the antitoxin degradation rate [15], [16]. The fluctuation of the Lon activity may be particularly important in determining switching rates, because it can provide coherent noise that favours simultaneous switching of many TAs to the persister state. It should also be noted that the Lon activity is activated by polyphosphate, which is regulated by the stringent response signalling molecule (p)ppGpp [16]. We plan to extend the present model to include these features and study the switching behavior in near future.

It is still interesting to think about possible implication of the observed switching rate to the present model. The fact that the persister formation is a rare event may indicate that the actual parameter value in the real system is located close to the boundary between the bistable region and the monostable region of the antitoxin-dominated state. Such parameter values can be chosen through selection process in a fluctuating environment, where slow growth of the persister pays off as a risk hedging strategy; the switching rate is expected to reflect the time scale of the temporal fluctuation of the environment [29].

**Figure 5:**
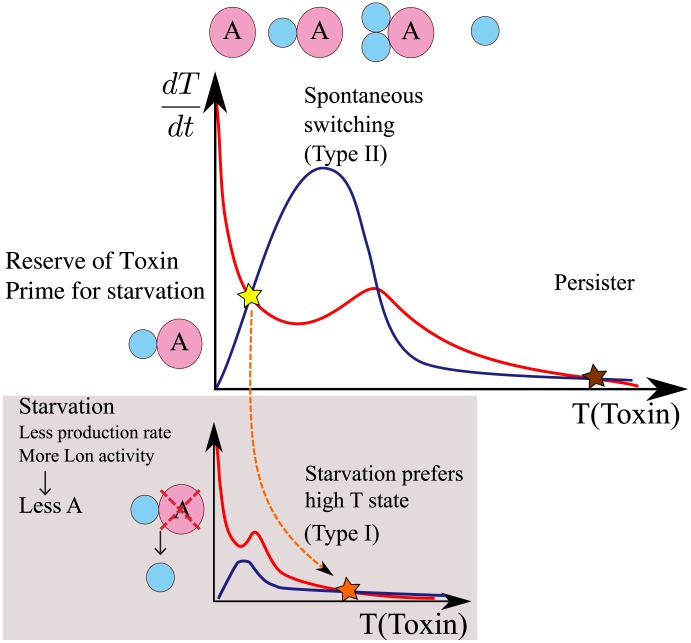
Schematic summary of the role of conditional regulation in persister formation. The red curves show the toxin production rate and the blue lines give the degradation rate, both from eq. (6). Both terms depend on 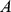, and here we make approximation that 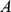 is always in steady state (eq. 7 with 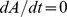) for given 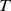, because dynamics of 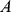 is much faster than 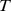 due to high production and degradation rate. Since production term of 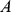 and 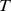 are proportional to each other and 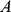 is degraded at a constant rate, resulting 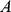 concentration is proportional to the production term of 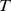 (red curves). The scales of curves are modified from actual functional forms so that the characteristic behaviours can be grasped easily. The ultra-sensitivity mediated by protein-protein binding combined with feedback from free toxin activity is reflected in the peak of the production rate and drop of the degradation rate, resulting in bistability of the system. This accounts for the type II persister where a cell can spontaneously switch to and out of the persister state. The non-monotonicity of the conditional regulation secures that some toxins are stored in antitoxin dominated state, helping the transition to the stress-induced activation of toxin [6], which becomes the base for type I persister formation.

Conditional regulation is an example of mixed feedback motifs [30], where protein-protein interactions and transcriptional repression are combined. In natural systems, protein-protein interaction mediated bistable switch was previously found for example in the epigenetic switch of the TP901 phage [23], [25] and in the sigma-factor/antisigma-factor system [24]. Conditional cooperativity in TA systems opens for a toolbox of regulatory units that can exhibit sufficient bistability to support also epigenetics. When removing the toxic ability of toxin, which has been done for RelE [3], and separating antitoxin from the operon to allow independent control, the strong binding between RelE and RelB should provide extreme ultrasensitivity, and thus very well separated metastable states. This conditional cooprativity-mediated bistability is the base for the bistability in full TA systems, and thus for the type II persister formation [12], [13], where a cell can spontaneously switch between the dormant state and the growing state (Fig. 5).

While simple protein-protein heteromers could produce ultrasensitivity, the non-monotonicity of the conditional cooperativity also secure that the antitoxin dominated state has a substantial amount of toxins present (Fig. 5). These toxins' activity is normally inhibited by short lived antitoxins, but the stored toxins can be used for faster switching to a dormant state if overall protein productions are externally inhibited, for example by starvation (Fig. 5). Therefore, the non-monotonicity may enhance the transition to type I persister formation [12], [13], where environmental stress triggers persister formation.

The importance of the protein-protein interaction mediated ultrasensitivty [22]–[25] and the growth rate-mediated feedback [17], [26]–[28] to bistable systems have been discussed as independent regulatory features in recent literature [31]. The uniqueness of the bistability in the TA system is that *it combines both of these mechanisms*. The need for combining these two mechanisms is closely associated with the fact that T and A are produced from the same operon, and thus are exposed to identical transcription regulation. Though it is difficult to get bistability with only one of the mechanisms [17], the TA system realizes a persister state by regulating the products of one operon through a combination of growth modulation and hetero-complex formation.

## Materials and Methods

### Numerical solutions of the model equations

All the numerical analyses are done using C++ codes developed by the authors. When necessary, 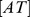 was calculated by solving algebraic equations (2) and (3) with conservation of mass for a given amount of 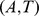 by Newton's method [32]. The bistable solutions in Fig. 2 B (Fig. 4) were obtained by finding the fixed points for 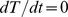 with eq. (4) (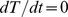 and 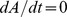 with eqs. 6 and 7) by Newton's method and then evaluating the stability based on the Jacobian. The trajectories that constitute the flux in Figs. 3A2 and 3B2 were calculated by the 4th-order Runge-Kutta method [32].

### Reference parameters

The values of the parameters used in the ODEs correspond to a conversion to dimensionless numbers of the parameters relative to the 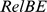 system we studied in [6].

In particular we used the lifetime of 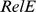 in exponential growth conditions (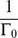) as time-unit (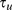) and the maximal amount of 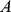 proteins produced in the unit time as concentration unit (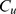). In the 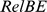 system 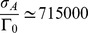 nM thus fixing 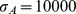 we obtain 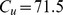 nM, while 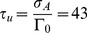 min. The value of 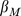 in the starved condition [6] was evaluated to be around 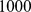 in this units. However, it is expected to be smaller in the normal condition, since RelE cleaves mRNA at the ribosomal A-cite, which is expected to be more accessible at the starvation. Therefore, we mostly explore 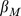 values smaller than 1000.

The reference parameters are shown in table 1.

**Table 1:** Reference parameter values.

	*X* [6]	→	→	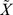
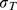	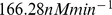	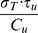	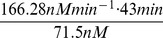	100
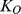	1 nM	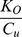	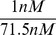	0.015
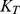	0.3 nM	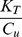	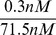	0.004
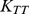	0.3 nM	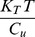	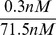	0.004
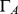	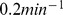	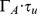	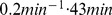	10
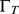	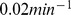	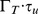	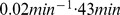	1
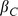	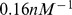	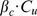	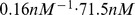	11
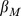	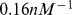	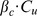	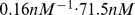	11

## Supporting Information

Figure S1
**Fit of the free toxin activity parameters to the grown-rate dependent global transcription rate.** Left: Red points: Global transcription rate 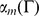 from Klumpp et al. [26]. Green Line: normalised production rate 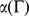 from our model with 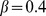. Right: Red points: Normalized global transcription rate multiplied by gene copy number, 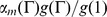 from Klumpp et al. [26]. Green Line: normalised production rate 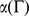 from our model with 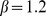.(EPS)

Figure S2

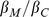
 **fitted to the global transcription rate lies in the bistable region.** Each green dot in the plot represents a combination of 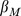 and 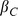 that give bistable results. The red line represents 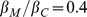, and and the black line 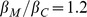.(EPS)

Figure S3
**The robustness of the bistability against parameter change.** We fix 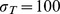 and 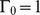, and vary rest of the parameters. In (a) 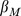 is changed systematically between 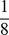 and 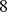 fold of the value used in the main text 
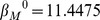
; we change it between 
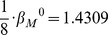
 and 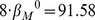 with a pace given by 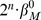 with an integer 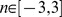. For each value of 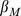, we sample rest of the parameters randomly and independently of each other, and they can take any values from the set 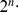
(the reference value) with 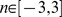. The reference values are given in Table 1. We collect a sample of 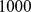 points in the parameter space. The bars in the histogram represent the fraction of this sample of points in the parameter space that still shows bistable behavior. The same procedure is then carried out for 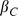 (b), 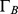 (c), 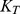 (d), 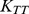 (e), 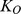 (f) and 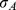 (g).(EPS)

Figure S4
**The robustness of the bistability against the change of the dissociation constants** 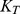 **and** 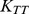 **.** We set 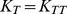, and increase them systematically from the reference value (0.004) to 64 fold of the reference value. Since the dissociation constants set the concentration of 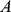 and 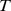 at which 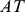 and 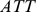 formation is significant, we fix 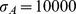 and 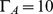 in addition to fixing 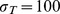 and 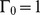. We then sample the rest of the parameters randomly in the base 2 logarithmic scale, within 1/8 to 8 fold of the reference value. We tried 1000 parameter sets for each values of 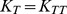. The plot shows the fraction of the parameter set that shows the bistability. We see that the number of bistability parameter sets decrease gradually with fold increase of the dissociation constants.(EPS)

Text S1 **Correspondence of parameters with the growth rate dependence data of protein production rate in the steady state growth.**
(PDF)

Text S2 **Parameter scan by Monte Carlo sampling to test the robustness of bistability.**
(PDF)
