# Supplementary material for: Conditional Cooperativity of Toxin - Antitoxin Regulation Can Mediate Bistability between Growth and Dormancy
Source: PLoS Comput Biol. 2013 Aug 29;9(8):e1003174. doi: 10.1371/journal.pcbi.1003174 (PMC3757081; doi:10.1371/journal.pcbi.1003174)
Supplement: Text S1 — Correspondence of parameters with the growth rate dependence data of protein production rate in the steady state growth. (PDF) [file pcbi.1003174.s005.pdf]

Supplement: Conditional cooperativity of Toxin - Antitoxin regulation can mediate bistability between growth and dormancy
Ilaria Cataudella, Kim Sneppen, Kenn Gerdes, and Namiko Mitarai

Text S1: Correspondence of parameters with the growth rate dependence data of protein production rate in the steady state growth

Klumpp et al. [1] reported that a decrease in cellular growth rate results in a global slow down of transcription, and provided the relative change of the transcription rate for steady state growth.

In our model equations,

$$\frac{dT}{dt} = \frac{\sigma_T}{\left(1+\frac{[AT]}{K_O}\right)(1+\beta_M[T_f])} - \frac{\Gamma_0}{1+\beta_C[T_f]}\cdot T \tag{1}$$

$$\frac{dA}{dt} = \frac{\sigma_A}{\left(1+\frac{[AT]}{K_O}\right)(1+\beta_M[T_f])} - \Gamma_A\cdot A. \tag{2}$$

the growth rate slows down due to the free toxin, characterised by β_C, while the protein production rate is reduced at the same time, which is characterised by β_M. We can reproduce the relation in [1] when β_C and β_M satisfy a certain relation as follows.

In our model, the term $\Gamma \equiv \frac{\Gamma_0}{1+\beta_C[T_f]}$ in equation is the division rate. In ref. [1], cell division rate is measured in terms of doubling per hour, while our Γ_0 is estimated to be 30 minutes doubling time [2]. Namely, we have

$$\Gamma = \frac{2dbl/hour}{1+\beta_C[T_f]}, \tag{3}$$

and we get the amount of free toxin as a function of Γ[dbl/hour]:

$$[T_f] = \frac{2-\Gamma}{\beta_C\cdot\Gamma}. \tag{4}$$

This gives the growth-rate dependent protein production change in our model to be

$$\tilde{\alpha}(\Gamma) = \frac{1}{1+\beta_M[T_f]} = \frac{\Gamma/(2\beta)}{1+\frac{1-\beta}{2\beta}\Gamma},$$

where $\beta \equiv \beta_M/\beta_C$.

In ref. [1], the growth-rate dependent change of the transcription rate $\alpha_m(\Gamma)$ normalised by the value at $\Gamma = 1$ and the gene copy number $g(\Gamma)$ is given. In Fig. S1 left, we plotted $\alpha_m(\Gamma)$ and fitted β so that normalised growth-dependent production rate

$$\alpha(\Gamma) = \frac{\tilde{\alpha}(\Gamma)}{\tilde{\alpha}(1)} = \frac{\frac{1+\beta}{2\beta}\cdot\Gamma}{1+\frac{1-\beta}{2\beta}\cdot\Gamma} \tag{5}$$

agrees best with the data. With $\beta \approx 0.4$, we get a good fit to the data.

If we also take into account the change in the gene copy number, we should compare $apha(\Gamma)$ with $\alpha_m(\Gamma)g(\Gamma)/g(1)$, which is shown in Fig. S1 right. In this case, we get $\beta \approx 1.2$.

In fig S2, we plotted the lines $\beta = \frac{\beta_M}{\beta_C} = 0.4$ and $\beta = \frac{\beta_M}{\beta_C} = 1.2$ in the phase diagram for the bistability with reference parameters, which demonstrate that we can get bistability with satisfying the relation from [1].

It should be noted that the growth-rate change in [1] is obtained by the steady state exponential growth, and the relation may not be the same if the growth rate is changed dynamically by the overproduction of toxin.

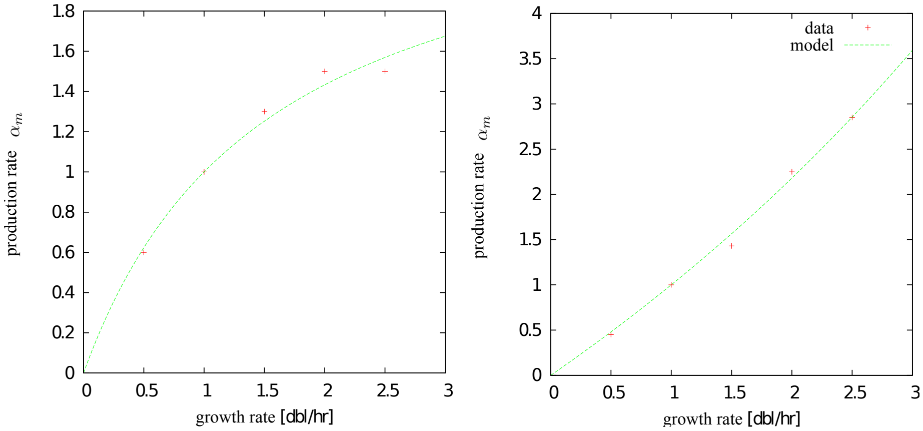

Figure S1: **Fit of the free toxin activity parameters to the grown-rate dependent global transcription rate.** Left: Red points: Global transcription rate $\alpha_m(\Gamma)$ from Klumpp et al. [1]. Green Line: normalised production rate $\alpha(\Gamma)$ from our model with $\beta = 0.4$. Right: Red points: Normalized global transcription rate multiplied by gene copy number, $\alpha_m(\Gamma)g(\Gamma)/g(1)$ from Klumpp et al. [1]. Green Line: normalised production rate $\alpha(\Gamma)$ from our model with $\beta = 1.2$.

References

[1] S. Klumpp, Z. Zhang, T. Hwa ”*Growth-rate dependent global effects on gene expression in bacteria*” Cell **139(7)** 1366-1375 (2009)

[2] I. Cataudella, A. Trusina, K. Sneppen, K. Gerdes and N. Mitarai *“Conditional cooperativity in toxin antitoxin regulation prevents random toxin activation and promotes fast translational recovery”* Nucleic Acids Res. **40** 6424-6434 (2012).

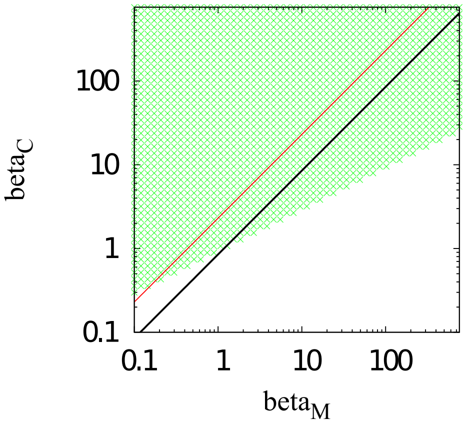

Figure S2: β_M/β_C **fitted to the global transcription rate lies in the bistable region.** Each green dot in the plot represents a combination of β_M and β_C that give bistable results. The red line represents $\beta_M/\beta_C = 0.4$, and and the black line $\beta_M/\beta_C = 1.2$.
