# Supplementary material for: Conditional Cooperativity of Toxin - Antitoxin Regulation Can Mediate Bistability between Growth and Dormancy
Source: PLoS Comput Biol. 2013 Aug 29;9(8):e1003174. doi: 10.1371/journal.pcbi.1003174 (PMC3757081; doi:10.1371/journal.pcbi.1003174)
Supplement: Text S2 — Parameter scan by Monte Carlo sampling to test the robustness of bistability. (PDF) [file pcbi.1003174.s006.pdf]

Supplement: Conditional cooperativity of Toxin - Antitoxin regulation can mediate bistability between growth and dormancy
Ilaria Cataudella, Kim Sneppen, Kenn Gerdes, and Namiko Mitarai

Text S2: Parameter scan by Monte Carlo sampling to test the robustness of bistability.

We tested the robustness of the bistabilty against parameter change by using the Monte-Carlo sampling. We fixed σ_T and Γ_0, which define the units, and scanned β_M, β_C, Γ_A, K_T, K_{TT}, K_O, and σ_A. In order to understand the systematic dependence on the parameter, if any, we change one of the parameters systematically, and sample the rest of the parameters randomly in the base 2 logarithmic scale, with in 1/8 to 8 fold of the reference value. We summarise the result in this section.

In fig S3 a) the effects of changing the value of β_M are investigated. The value used in the main text is ${\beta_M}^0 = 11.4475$ and we change it between $\frac{1}{8}{\beta_M}^0 = 1.4309$ and $8 \cdot {\beta_M}^0 = 91.58$. β_M quantifies the entity of the negative feedback on production of both A and T due to the increase in the concentration of T_f. The fraction of the sample of parameter sets that shows bistability decreases with increasing β_M. For high values of β_M bistability is lost because the high T fixed point tends to disappear. This is because an increase in T_f will result in a strong inhibition in production of both A and T, but T maximal production rate is , in the best case scenario, 10 times less than A's, thus the effect of the inhibition will be stronger on T, the rise in T_f will be counterbalanced and achieving a high T fixed point becomes harder.

Analogous reasoning can be carried out when looking at the effects of changing β_C in fig.S3 b). Again, $\beta_C^0 = 11.4475$ and is varied between $\frac{1}{8}{\beta_C}^0 = 1.4309$ and $8 \cdot {\beta_C}^0 = 91.58$. β_C quantifies the positive feedback on accumulation of T provided by increasing T_f (that slows down translation reducing frequency of cell division and thus degradation of T). Here the fraction of the sample of parameter sets that exhibits bistability tends to increase with increasing β_C, it peaks for $\beta_C \simeq 2 - 4 \cdot \beta_C^0$ and goes slightly down again at $8 \cdot \beta_C^0$. The reason for this behavior is the following: for low values of β_C an increase in T_f will not be sufficient to inhibit cell division enough to sustain the increase in T, thus it's hard to obtain a high T fixed point. As β_C increases it becomes easier and easier to achieve a high T fixed point, but if β_C becomes too high, a very small increase in T_f can be amplified to the point that it becomes harder and harder to sustain a low T fixed point, thus bistability is lost again for a higher fraction of parameter sets.

In Fig S3 c) the effect of changing the degradation rate for A is explored. The value of Γ_A used in the main text is $\Gamma_A^0 = 10$ and hereby we change it between $\frac{1}{8}{\Gamma_A}^0 = 1.25$ and $8 \cdot {\Gamma_A}^0 = 80$. The highest fraction of bistable set of

parameters is detected for the value of Γ_A used in the main text. Both for lower and higher values the bistabilty fraction decreases. In fact, for high values of Γ_A it becomes hard to obtain high A domniated fixed points. The toxin is degraded at the rate 1, so if Γ_A is low, considering the fact that A produced more than T, the system will in most cases (parameter sets) end up in a monostable high A state.

In Fig. S3 d) and e) the effect of changing respectively K_T and K_{TT} between $\frac{1}{8}K^0_{T(T)} = 0.0005$ and $8 \cdot {K_{T(T)}}^0 = 0.032$ ($K^0_{T(T)} = 0.004$) is investigated. The effect of changing K_T is practically irrelevant in this range, because the reference parameter is already in very strong binding limit for AT formation. Higher values of K_{TT}, on the other hand, results in a slightly lower fraction of bistable sets of parameters. As stated in the main text one of the key ingredient for achieving bistability is the protein sequestration mechanism, and in particular, the resulting ultrasensitive behavior. High values of K_{TT} (weak binding) will weaken ultrasensitivity, resulting in a decrease in the bistable fraction.

In Fig S3 f) we study the effect of changing the binding constant of the repression factor AT to the operator. The value used in the main text for K^0_O is 0.015. Thus we explore the behavior of the system for values of K_O ranging between $\frac{1}{8}K^0_{T(T)} = 0.001875$ and $8 \cdot {K_{T(T)}}^0 = 0.12$. The fraction of bistable parameter set increases with increasing K_O. This is because if the binding of AT complexes to the operon region is very tight, a low concentration of AT is enough to keep the promoter repressed all the time, making the A dominated state, which requires high production of A due to high degradation rate, difficult to maintain.

Finally we investigate the robustness against change of the value of σ_A. As in can be seen in fig S3 (g), the consequences are not dramatic within this range, but it is evident that for low values of σ_A, the production advantage with respect to T becomes insufficient to compensate for high degradation rate for A, thus for many parameter combinations it is hard to obtain a high A fixed point. In the main text, we show systematic dependence on σ_A/σ_T for wider range.

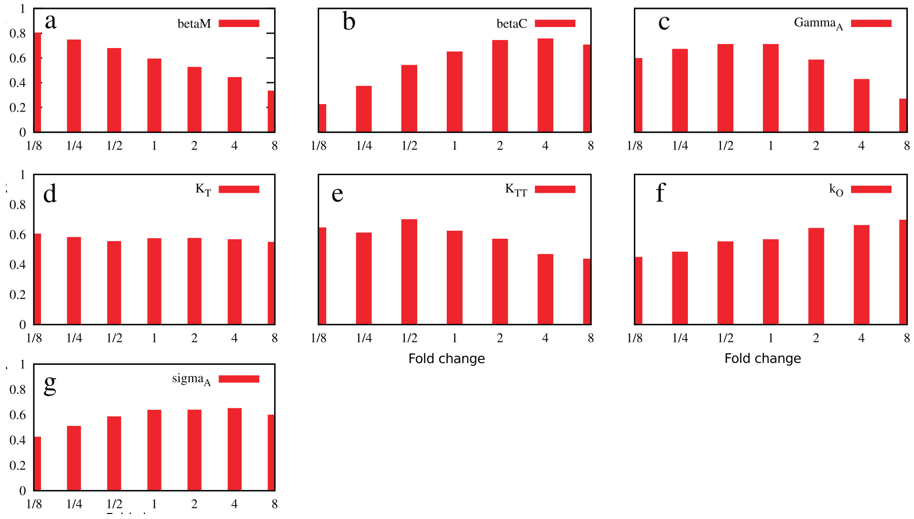

Figure S3: **The robustness of the bistability against parameter change.** We fix $\sigma_T = 100$ and $\Gamma_0 = 1$, and vary rest of the parameters. In (a) β_M is changed systematically between $\frac{1}{8}$ and 8 fold of the value used in the main text ${\beta_M}^0 = 11.4475$; we change it between $\frac{1}{8} \cdot {\beta_M}^0 = 1.4309$ and $8 \cdot {\beta_M}^0 = 91.58$ with a pace given by $2^n \cdot \beta_M^0$ with an integer $n \in [-3, 3]$. For each value of β_M, we sample rest of the parameters randomly and independently of each other, and they can take any values from the set $2^n \cdot$(the reference value) with $n \in [-3, 3]$. The reference values are given in Table **??**. We collect a sample of 1000 points in the parameter space. The bars in the histogram represent the fraction of this sample of points in the parameter space that still shows bistable behavior. The same procedure is then carried out for β_C (b), Γ_B (c), K_T (d), K_{TT} (e), K_O (f) and σ_A (g).

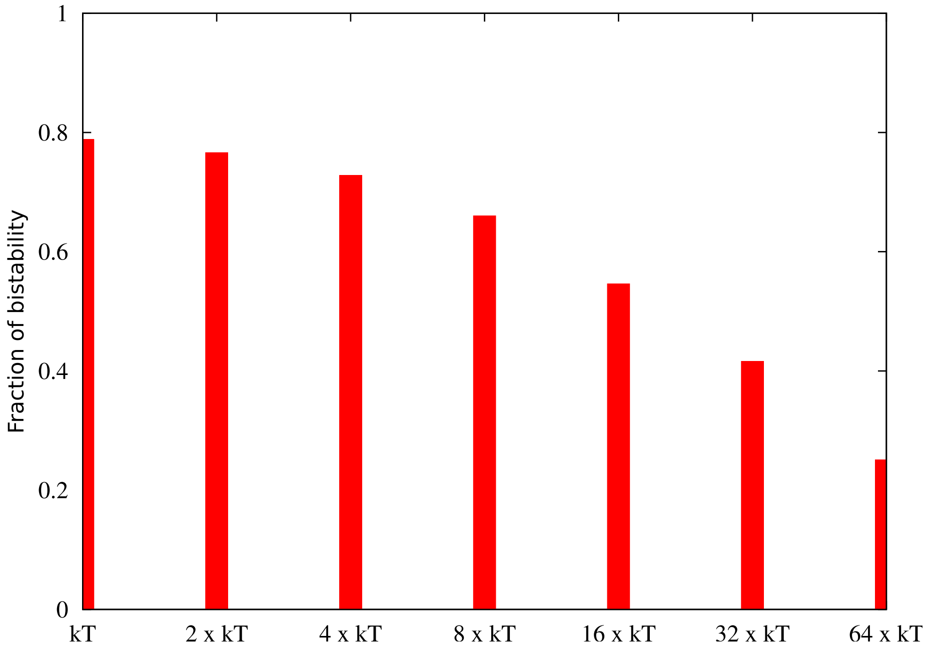

Figure S4: **The robustness of the bistability against the change of the dissociation constants K_T and K_{TT}.** We set $K_T = K_{TT}$, and increase them systematically from the reference value (0.004) to 64 fold of the reference value. Since the dissociation constants set the concentration of A and T at which AT and ATT formation is significant, we fix $\sigma_A = 10000$ and $\Gamma_A = 10$ in addition to fixing $\sigma_T = 100$ and $\Gamma_0 = 1$. We then sample the rest of the parameters randomly in the base 2 logarithmic scale, within 1/8 to 8 fold of the reference value. We tried 1000 parameter sets for each values of $K_T = K_{TT}$. The plot shows the fraction of the parameter set that shows the bistability. We see that the number of bistability parameter sets decrease gradually with fold increase of the dissociation constants.
